# Correlation Between the Neutrophil-to-Lymphocyte Ratio and the Severity of Coronary Artery Disease in Patients With Myocardial Infarction

**DOI:** 10.7759/cureus.69061

**Published:** 2024-09-10

**Authors:** Nadeem Ahmad, Muhammad Tahir Raza, Muhammad Ammar Ali, Urooj Tahir, Hamza Ibrahim, Ahmad S Iqbal, Rana Shahzaib Ali, Muhammad Arslan Tariq, Saqib Majeed, Muhammad Hassan, Sana Liaquat, Tayyab Mumtaz Khan

**Affiliations:** 1 Cardiology, Allama Iqbal Medical College, Lahore, PAK; 2 Cardiology, Rawalpindi Medical University, Rawalpindi, PAK; 3 Medicine, Rawalpindi Medical University, Rawalpindi, PAK; 4 Medicine, Allama Iqbal Medical College, Lahore, PAK; 5 Cardiology, Sheikh Zayed Medical College and Hospital, Rahim Yar Khan, PAK; 6 Emergency, University Hospital Coventry and Warwickshire, Coventry, GBR; 7 Orthopaedic Surgery, Rawalpindi Medical University, Rawalpindi, PAK

**Keywords:** artery, coronary, correlation, disease, infarction, lymphocyte, myocardial, neutrophil, ratio

## Abstract

Background

Coronary artery disease (CAD) is one of the major causes of death all over the world. Its severity evaluation through the Gensini scoring system is quite a complex process as these score systems require complex investigations and cardiologists. Therefore, this study aimed to determine the predictive capacity of neutrophil-to-lymphocyte ratio (NLR) for the severity of CAD in patients with myocardial infarction.

Methods

This cross-sectional study was performed on 208 patients with acute myocardial infarction whose coronary angiography was performed in the Department of Cardiology of Benazir Bhutto Hospital (BBH), Rawalpindi, Pakistan during the period of one year from January 2022 to March 2023. The enrollment of patients was made through purposive sampling and established inclusion and exclusion criteria. Ethical approval and informed consent were acquired before the data collection. Data was collected through a self-structured form. Vessel score and Gensini score were applied for the assessment of the severity of CAD. Patients were divided into two groups based on the Gensini score system. Data analysis was carried out in the Statistical Package for the Social Sciences (SPSS) version 25 (Released 2017; IBM Corp., Armonk, New York, United States). Both descriptive and inferential statistics were used to compare the study variables between the patients with non-severe CAD and patients with severe CAD. Pearson’s correlation was used to determine the correlation between the NLR and the severity of CAD. A linear regression model was applied to evaluate the predictive capacity of the NLR for the severity of CAD. A p-value less than 0.05 was set as statistically significant.

Results

Out of 208 patients, 128 (61.53%) patients had non-severe CAD while 80 (38.47%) patients had severe CAD. Significant differences were observed in the Gensini mean scores, NLR values, and in the frequencies of hypertension, diabetes mellitus, dyslipidemia, and history of smoking, with p≤0.05 for all these variables, between the non-severe CAD group and severe CAD group. NLR was significantly correlated with the severity of CAD (p-value=0.001). Pearson’s correlation coefficient was 0.71 for NLR with the Gensini scores. The simple linear regression model was valid (the p-value of the F test was <0.000), with beta coefficients of 2.60 (p=0.002) for NLR. The R^2^ value was 0.80 (80%).

Conclusions

In the current study, a significant percentage of patients had severe CAD. Furthermore, a positive and significant association was noted between the NLR with the severity of CAD. This present study suggests that NLR is a reliable predictor of CAD severity; therefore, it could be used for risk stratification of cardiac patients with CAD.

## Introduction

Coronary artery disease (CAD) is the leading cause of morbidity and mortality across the world. It is estimated that only CAD caused almost 17.9 million deaths in 2019 in the world. It has contributed to 32% of all deaths and 80% of those deaths occurred mainly in developing countries. This number is expected to reach 23.6 million in 2030 [1,2]. In Pakistan, the prevalence of coronary heart disease (CHD) is also high (15.31% to 18.90%) like in other South Asian countries. In 2019, according to a Global Burden of Disease study, the approximated age-standardized incidence of cardiovascular was higher in Pakistan (918.18/100,000) than rest of the world (684.33/100,000) [3,4].

Based on various clinical presentations of CAD, it has been classified into acute coronary syndrome (ACS) and chronic coronary syndromes/stable ischemic heart disease. ACS is also further divided into three groups including non-ST-elevation MI (NSTEMI), unstable angina (UA), and ST-elevation MI (STEMI). Acute myocardial infarction (MI) is the worst form of CAD and includes both STEMI and NSTEMI [1,5]. The prevalence of acute MI is also higher in South Asian countries. In Pakistan, 30% of its population with age above 45 years are affected by this cardiovascular disease [6].

Atherosclerosis plays a key role in the pathophysiology of CAD. Atherosclerosis is an inflammatory condition. Inflammation is associated with the development, progression, and destabilization of the atherosclerotic plaque in the vessels. Several studies have shown a strong positive correlation between inflammatory markers and cardiovascular events among patients with CAD [2,7]. Different inflammatory markers have been used as biomarkers of cardiovascular diseases and some important of those are the following: interleukin-6 (IL-6), monocyte colony-stimulating factor (MCSF), IL-1 isoform, fibrinogen, C-reactive protein (CRP), highly sensitive CRP, tumor necrosis factor-alpha (TNF-α), and lipoprotein-associated phospholipase A2 [1,8].

The neutrophil-to-lymphocyte ratio (NLR), which is calculated by dividing the number of neutrophils by the number of lymphocytes from same blood sample, has become apparent as a new inflammatory marker of adverse cardiovascular events. Its normal value is approximately between 0.78 and 3.53 [9]. During inflammation, various changes take place in the white blood cells, especially in neutrophils and lymphocytes. The NLR indicates the balance between innate immunity via neutrophil count and adaptive immunity through lymphocyte count during inflammation. Neutrophil is known to exacerbate the inflammatory process during atherosclerosis by releasing different inflammatory mediators (chemokines and cytokines). Whereas lymphocytes suppress inflammation and consequently, the incidence of both atherosclerosis and CAD also goes down. In literature, it has been reported in various studies that higher NLR is associated with a higher incidence of adverse cardiac events [10-12].

Around the globe, the predictive role of NLR for cardiovascular events has been demonstrated in various studies [1,2,7-12]. However, in Pakistan, studies on the association between the NLR and the severity of CAD are lacking. Therefore, this study aims to determine the correlation between the NLR and the severity of CAD in patients with acute MI.

## Materials and methods

Study design and study population

This cross-sectional study was performed in the Department of Cardiology of Benazir Bhutto Hospital (BBH), Rawalpindi, Pakistan during the period of one year from January 2022 to March 2023. Two hundred and eight patients with acute MI whose coronary angiography was performed were recruited in the study via purposive sampling technique and established inclusion and exclusion criteria. For the current study, the sample size was calculated by the WHO sample size calculator. During the sample size calculation, we kept the power of the study at 80% while the confidence interval and margin of error were 95% and 5%, respectively.

Inclusion and exclusion criteria

All patients with either gender, age 40 years or above, and complete medical history regarding ACS (diagnosed via electrocardiograph, cardiac biomarkers, and coronary angiography for accuracy) were included in the study. While patients with age below 40 years, a history of previous percutaneous coronary intervention (PCI), coronary artery bypass graft surgery (CABG), heart failure (New York Heart Association (NYHA) classes III, IV), congenital heart disease, chronic liver disease, chronic kidney disease, autoimmune disease, active infectious disease, oncological conditions, and who were not willing to participate were excluded from the study.

Ethical approval

Ethical approval was obtained from the Ethical Review Board (ERB) of BBH, Rawalpindi, Pakistan. The ethical approval number was BBH.ERB.283/267. Written informed consent was also acquired from each participant after the proper elaboration of the study. BBH is an allied hospital of Rawalpindi Medical University, Rawalpindi.

Acute MI

The diagnosis of MI was made according to the criteria for MI of the American Heart Association. It was based on severe chest pain of 30 minutes duration, specific electrocardiographic (ECG) patterns of MI, and significant elevation in cardiac enzymes [1].

Severity of CAD and NLR

According to the American College of Cardiology/American Heart Association lesion classification, the presence of severe stenosis, at least 50% of the vessel diameter, in any of the primary coronary arteries was taken as CAD [13]. It was diagnosed by coronary angiography. Its severity was diagnosed by using the Gensini score and multiplier factor of vessel score. The Gensini score was determined according to the percentage of stenosis of the lumen of the coronary arteries as follows: 1 for 1% to 25%, 2 for 26% to 50%, 4 for 51% to 75%, 8 for 76% to 90%, 16 for 91% to 99%, and 32 for total occlusion. The vessel score was used as a multiplier for the Gensini score and then the total score was calculated. Vessel score was assessed by the location of the lesions as follows: 0.5 for the second diagonal and second obtuse marginal branches; 1 for the distal segment of left anterior descending (LAD) and left circumflex (LCX), first diagonal branch, first obtuse marginal branch, right coronary artery, posterior descending artery, and intermediate artery; 1.5 for the mid-segment LAD and LCX; 2.5 for the proximal LAD or LCX; and 5 for the left main lesion. The total Gensini score is equal to the sum of the score for the percentage of stenosis multiplied by the score for vessel(s). Using the Gensini scoring system, patients with coronary stenosis were split into two groups: patients with a Gensini score up to 50 were labeled to have non-severe CAD while patients with a Gensini score above 50 were described as having severe CAD [1]. From the peripheral blood sample, the NLR was calculated by dividing the number of neutrophils by the number of lymphocytes.

Data collection

A self-designed proforma was applied for data collection. It had two components. The first was about history and examination findings such as age, gender (male or female), and the presence or absence of conventional risk factors of CAD (hypertension, diabetes mellitus, dyslipidemia, and history of smoking). The second component was about the reports of all investigations’ reports. All investigations including electrocardiogram (ECG), cardiac biomarkers, coronary angiography, complete blood count for the calculation of NLR, and serum lipid level, were performed at BBH during patients’ management.

Data analysis

IBM SPSS Statistics for Windows, Version 25 (Released in 2017; IBM Corp., Armonk, New York, United States) was used for statistical analysis in this study. Quantitative data were shown through mean ± standard deviation whereas the qualitative data were presented via frequencies and percentages. Independent samples t-tests and chi-squared were used to compare the numerical and nominal study variables between the two study groups respectively. Pearson's correlation analysis was applied to assess the direction and strength of the association between NLR ratio and Gensini scores. The predictive capacity of NLR for Gensini scores was evaluated by the linear regression model. A p-value of less than 0.05 was viewed as statistically significant.

## Results

Out of 208 patients, 128 (61.53%) had non-severe CAD while 80 (38.47%) patients had severe CAD according to the Gensini score system. The means of study variables such as age, Gensini score, and the NLR value were 57.50 years with an SD of ±15.23, 48.42 with an SD of ±35, and 5.08 with an SD of ±2.80, respectively.

Table 1 elaborates on the characteristics of the study population. Statistically significant differences were noted in the Gensini mean scores, NLR values, frequencies of hypertension, diabetes mellitus, dyslipidemia, and history of smoking, with p<0.05 for all these variables, between the non-severe CAD group and the severe CAD group of patients. However, there were no significant differences present between the non-severe CAD group and the severe CAD group, in the age and gender, with p>0.05 for these variables.

**Table 1:** Demographic characteristics of study population along with independent t-test and Chi-squared test analysis of study variables MI: myocardial infarction; NLR: neutrophil-to-lymphocyte ratio; CAD: coronary artery disease; SD: standard deviation p-values with the sign of (+) are of independent t-tests while p-values with the sign of (*) are of Chi-square test.

Variables of MI Patients (N=208)		Presentation of Variables	Status of CAD		Independent t-test/Chi-Square Test
			Non-severe CAD Group n=128 (61.53%)	Severe CAD Group n=80 (38.47%)	p-values
Age (Years) (Means ± SD)		57.50 ± 15.23	55.12 ± 12.75	59.14 ± 15.45	0.07^+^
Gensini Score (Means ± SD)		48.42 ± 35.00	28.43 ± 18.10	70.22 ± 33.67	0.001^+^
NLR Value (Means ± SD)		5.08 ± 2.80	1.70 ± 0.50	3.90 ± 2.60	0.001^+^
Gender	Male n (%)	138 (66.36)	80 (62.50)	58 (72.50)	0.08^*^
	Female n (%)	70 (33.64)	48 (37.50)	22 (27.50)	
Hypertension	Yes n (%)	157 (75.48)	88 (68.75)	69 (86.25)	0.02^*^
	No n (%)	51(24.52)	40 (31.25)	11 (13.75)	
Diabetes Mellitus	Yes n (%)	134 (64.42)	69 (53.90)	65 (81.25)	0.01^*^
	No n (%)	74 (35.58)	59(46.10)	15 (18.75)	
Dyslipidemia	Yes n (%)	124 (59.61)	68 (53.13)	56 (70.00)	0.03^*^
	No n (%)	84 (40.39)	60 (46.87)	24 (30.00)	
History of Smoking	Yes n (%)	107 (51.44)	52 (40.63)	55 (68.75)	0.04^*^
	No n (%)	101 (48.56)	76 (59.37)	25 (31.25)	

Table 2 shows the direction and strength of the correlation between the NLR ratio and the severity of CAD in the study population through Pearson’s correlation analysis. The correlation coefficient was positive and statistically significant. A positive correlation coefficient means with the rise in the Gensini score, the NLR value will also increase.

**Table 2:** Correlation between NLR ratio and severity of CAD in study population NLR: neutrophil-to-lymphocyte ratio; CAD: coronary artery disease; SD: standard deviation

Variables N=208	Severity of CAD		Pearson’s Correlation	
	Non-severe CAD Group n=128 (61.53%)	Severe CAD Group n=80 (38.47%)	Correlation Coefficient (r)	P-value
NLR Value (Means±SD)	1.70±0.50	3.90±2.60	0.71	0.001

Table 3 indicates the findings of the simple linear regression model, which was a valid model in the current study, as the F-test was greatly significant with p-value <0.000. The R^2^ value was also highly significant at 0.80 (80%). The beta coefficient was positive for NLR, with a statistically significant p-value. A positive beta coefficient means the higher the NLR value, the higher the Gensini score, and consequently, CAD would be more severe.

**Table 3:** Linear regression model for study variables R^2^ value was 0.80 (80.00%). The regression model was significant as the p-value of the F-test was <0.000 (significant). NLR: neutrophil-to-lymphocyte ratio; CI: confidence interval

Variable	Unstandardized Regression Coefficient (B)	95% CI	p-value
NLR Value	2.60	1.44 to 4.25	0.002

## Discussion

In this current study, we have obtained valuable information about the correlation of the NLR with the severity of CAD. Furthermore, we have also observed the incidences of various conventional risk factors of CAD and differences in the frequencies of these risk factors between the two study groups such as patients with non-severe CAD and patients with CAD.

In the current study population, out of 208 patients, 128 (61.53%) had non-severe CAD while 80 (38.47%) patients had severe CAD. Higher incidences of non-severe CAD and severe CAD have been noted in the Turkish population. This difference in the frequencies of non-severe CAD and severe CAD could be due to the variation in the frequencies of conventional risk factors among these two studies’ populations [14].

Regarding the demographic features of the study population, it was noted that the mean age in patients with severe CAD (59.14 with SD of ±15.45 years) was higher than in patients with non-severe CAD (55.12 with SD of ±12.75 years). Similarly, male gender (n=138, 66.36%) was dominant in the study participants. Another study has also reported alike findings about the demographic characteristics of the patients with CAD [1].

The most prevalent conventional risk factor in the current study was hypertension (n=157, 75.48%), followed by diabetes mellitus (n=134, 64.42%), dyslipidemia (n=124, 59.61%), and smoking (n=107, 51.44%). Significant differences in the frequencies of these risk factors were noted between the two study groups (p=less than 0.05). Various studies have endorsed the results of our study about the incidences of these risk factors and differences in these risk factors between the two study groups such as patients with non-severe CAD and patients with severe CAD, as those studies had found similar results [15,16].

The main aim of this study was to investigate the correlation between NLR and the severity of CAD in patients with acute MI. A statistically significant association between NLR and CAD severity was noted by Pearson’s correlation with a correlation coefficient (r) of 0.71 and p-value of 0.001. Patients with severe CAD had higher NLR values compared to those with non-severe CAD. The simple linear regression model further supports this association, with NLR being a significant predictor of CAD severity with a p-value of 0.002. Different studies all over the world have supported this finding of the current study about the correlation between NLR and the severity of CAD. An American study has also demonstrated the same results about the association of NLR and CAD severity [7]. Similarly, another study from Romania has presented a significant association between NLR and CAD [10]. A Chinese study has also shown consistent results regarding NLR and CAD severity. It has manifested that with an increase in the severity of CAD, the value of NLR also goes up [11]. Another study from Indonesia has also revealed that patients with severe CAD have a higher NLR ratio in contrast to patients with mild to moderate CAD [12]. A Turkish study has noted the difference in NLR value in the different groups of patients based on CAD severity [14]. In light of previous studies and their endorsement of the results of the current study, it could be assumed that NLR is a simple and reliable biomarker of the severity of CAD.

The potential mechanisms underlying this association are not fully understood but may involve the role of inflammation in atherosclerosis and consequently CAD. Neutrophils and lymphocytes play important roles in the inflammatory process. An imbalance between these cell types may contribute to the development and progression of CAD as neutrophils has pro-inflammatory while lymphocyte has ant-inflammatory role. Neutrophil causes inflammation and consequent vessel wall damage by releasing the inflammatory mediators. While lymphocyte stops the inflammatory process [1,5]. In literature, the recently published work of the last few years about the association of NLR and CAD has pointed towards a specific mechanism of high NLR in patients with CAD. According to this mechanism a variety of chronic diseases, such as cancer, hypertension, and heart disease, induce bone marrow to release the granulocytic myeloid-derived suppressor cells (gMDSCs). These cells are immunomodulatory, and it is well-recognized that these cells reduce the anti-inflammatory lymphocyte response. Therefore, it is logical to assume that the NLR is an estimate of the phenotypic activity of gMDSCs that are released from bone marrow in response to damage caused by chronic diseases. As a result, it is also suitable to suppose that the rise in neutrophils count because gMDSCs released from the bone marrow can raise about 10% of the peripheral blood leukocytes. At the same time, their regulatory action simultaneously reduces the number of lymphocytes in peripheral blood [7,17]. This mechanism has elaborated the association of NLR with the severity of CAD. When the CAD worsens, more gMDSCs are released from bone marrow which causes a rise in neutrophil count while reducing lymphocyte count ultimately leads to an increase in NLR among patients with CAD.

Our study has several implications for clinical practice. NLR is a readily available and cost-effective biomarker that can be easily calculated from routine blood tests. Its inclusion in cardiac risk stratification may help identify patients at higher risk of adverse cardiovascular events. Additionally, patients with higher NLR values may benefit from more aggressive management and closer monitoring. So, our study suggests that NLR is a useful biomarker for predicting CAD severity in patients with acute MI. Its simplicity and cost-effectiveness make it an attractive tool for cardiac risk stratification, and further research is warranted to explore its potential clinical applications.

Limitations of the study

Along with the implications of the current study, this study also has some limitations. The cross-sectional design and single-center setting may limit the generalizability of our findings. Furthermore, a small sample size of the current study could also hinder the general applicability of the current study results. Therefore, further studies with different study designs and in divergent locations are needed to confirm these results and explore the potential mechanisms underlying the association between NLR and CAD severity.

## Conclusions

In the current study, patients with angiographically severe CAD had higher NLR in contrast to the patients with non-severe CAD. NLR was positively and significantly correlated with the severity of the CAD through the Gensini scoring system. Furthermore, the procedure of calculation of NLR is simple and cost-effective. Therefore, it should be included in cardiac risk stratification of the patients with CAD especially in the health centers where advanced coronary investigations such as coronary angiography are not available. Our study also recommends that cardiac patients with higher NLR should be managed promptly and adequately by cardiologists as they have more severe CAD than patients with lower NLR.
